# Electronic Flat Band in Distorted Colouring Triangle Lattice

**DOI:** 10.1002/advs.202303483

**Published:** 2023-10-15

**Authors:** Yaqi Li, Shuwei Zhai, Yani Liu, Jingwei Zhang, Ziyuan Meng, Jincheng Zhuang, Haifeng Feng, Xun Xu, Weichang Hao, Miao Zhou, Guang‐Hong Lu, Shi Xue Dou, Yi Du

**Affiliations:** ^1^ School of Physics Beihang University Haidian Beijing 100191 China; ^2^ Centre of Quantum and Matter Sciences International Research Institute for Multidisciplinary Science Beihang University Beijing 100191 China; ^3^ Institute of Physics Chinese Academy of Sciences Beijing 100190 China; ^4^ Institute for Superconducting and Electronic Materials, Australian Institute for Innovative Materials University of Wollongong Wollongong New South Wales 2500 Australia; ^5^ Beihang Hangzhou Innovation Institute Yuhang Hangzhou 310023 China; ^6^ Beijing Key Laboratory of Advanced Nuclear Materials and Physics Beihang University Beijing 100191 China; ^7^ Institute of Energy Materials Science University of Shanghai for Science and Technology Yangpu Shanghai 200093 China

**Keywords:** colouring triangle lattice, flat band, frustrated lattice, potassium, scanning tunneling microscopy

## Abstract

Dispersionless flat bands (FBs) in momentum space, given rise to electron destructive interference in frustrated lattices, offer opportunities to enhance electronic correlations and host exotic many‐body phenomena, such as Wigner crystal, fractional quantum hall state, and superconductivity. Despite successes in theory, great challenges remain in experimentally realizing FBs in frustrated lattices due to thermodynamically structural instability. Here, the observation of electronic FB in a potassium distorted colouring triangle (DCT) lattice is reported, which is supported on a blue phosphorene‐gold network. It is verified that the interaction between potassium and the underlayer dominates and stabilizes the frustrated structures. Two‐dimensional electron gas is modulated by the DCT lattice, and in turn results in a FB dispersion due to destructive quantum interferences. The FB exhibits suppressed bandwidth with high density of states, which is directly observed by scanning tunneling microscopy and confirmed by the first‐principles calculation. This work demonstrates that DCT lattice is a promising platform to study FB physics and explore exotic phenomena of correlation and topological matters.

## Graphical abstract

This paper reports a distorted colouring triangular lattice (DCT) synthesized with potassium atoms deposited on a two‐dimensional blue phosphorene‐gold network on Au(111) and use scanning tunneling microscopy and spectroscopy to investigate the structural and electronic flat band properties of the assembled DCT lattice. This work provides a promising platform for realizing flat‐band caused strongly correlated physics in self‐assembled DCT lattice.

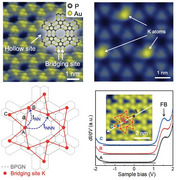

## 1 Introduction

The dispersionless band near Fermi level in momentum space, namely a flat band (FB), has attracted intensive attentions as it paves a way to lead to exotic phenomena originated from many‐body interaction, such as high‐ temperature fractional quantum Hall effect,^[^ ^1^, ^2^, ^3^ ^]^ Wigner crystal,^[^ ^4^ ^]^ superconductivity^[^ ^5^, ^6^, ^7^, ^8^, ^9^, ^10^ ^]^ and magnetism.^[^ ^11^, ^12^ ^]^ Recently, FB electronic states have been theoretically proposed in a few works by constructing and/or manipulating two‐dimensional (2D) frustrated lattices, including kagome,^[^ ^13^ ^]^ Lieb,^[^ ^14^ ^]^ and colouring‐triangle (CT) lattices.^[15^ ^]^ In these lattices, free electrons are confined in 2D space and commonly possess a completely quenched kinetic energy as a result of the destructive interference of wave functions in certain energy range. Consequently, the FB with strong electron correlations forms when the Coulomb interaction exceeds electron kinetic energy.^[16^ ^]^ Even though the theoretical predictions of FB have long been hugely attractive and inspired, experimental realization of these lattices remains extremely challenging because of their structural instability.^[^ ^17^, ^18^, ^19^ ^]^ CT electronic lattice, for instance, is originated from hexagonal lattice with parts of nearest‐neighbor (NN) hopping blocked.^[^ ^15^, ^20^ ^]^ The 2D electron gas (2DEG) in CT lattice experiences quantum interference at different lattice sites, which eventually causes the localization of electrons surrounding the forbidden hopping paths. The FB would be formed as the blocked NN hopping can be achieved by precisely increasing the distance between the corresponding NN lattice sites, which eventually causes a distorted colouring‐triangle (DCT) lattice.

Recently, 2D blue phosphorene‐gold network (BPGN) on Au(111) has been experimentally obtained in which 2DEG of the metallic substrate is reserved.^[^ ^21^, ^22^, ^23^ ^]^ Abundant bridging sites, the middle of two adjacent gold atoms in BPGN, form periodic frustrated structures, such as kagome and DCT configurations.^[24^ ^]^ In quantum destructive principle, 2DEG system with parabolic energy dispersion can be transformed to FB system if it is modulated by a potential field with as‐mentioned frustrated geometries. However, local electroneutrality of BPGN bridging sites limits their capability in modulating the energy dispersion of 2DEG. In previous work, we showed potassium (K) atoms can reside and move over various hollow sites, which are in the center of hexagonal P_9_ subunits in BPGN.^[25^ ^]^ With local positive potentials they can induce the variation of local density of states. It inspires us that the frustrated potential lattice can be constructed and stabilized by anchoring K atoms at the bridging sites with a desired coverage on BPGN. Then, the energy dispersion of 2DEG is expected to transform into FBs when the criteria of electron hopping meet.

Here, we experimentally construct an electronic DCT lattice by arranging K atoms over bridging sites on BPGN, in which the energy dispersion of 2DEG is modulated into FBs. We have demonstrated that the specific frustrated potential fields and electronic hopping strength can be feasibly tuned by controlling the coverage and resident sites of K atoms. Moreover, an electronic FB was measured in entire DCT lattice by scanning tunneling spectroscopy (STS) as a pronounced peak, which was further validated by a combination of tight‐binding (TB) model and first‐principles calculations on the frustrated DCT lattice. Our work paves a way for creating electronic FBs by constructing stable frustrated potential field and offers a feasible access to explore exotic properties in FB systems by manipulating electronic hopping strength.

## 2 Results and Discussion

**Figure 1:**
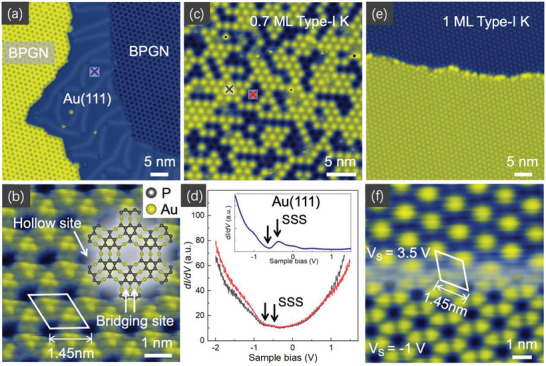
2DEG on the BPGN and Type‐I K substrate. a) Large‐scale STM images of the BPGN on Au(111) (V_S_ = −0.9 V, I = 400 pA). b) High resolution STM image in a flatten BPGN surface. The white rhombus marks the unit cell of the 4 × 4 BPGN superstructure (V_S_ = −1.0 mV, I = 50 pA). The hollow site and bridging site are indicated by white arrows. The atomic structure is placed on the image to depict the sites of different atoms. c) BPGN with 0.7 ML K deposited on the hollow sites (V_S_ = 3.5 V, I = 50 pA). d) d*I*/d*V* spectra collected on the surface of partially covered BPGN layers. Grey and red lines represent the data acquired at the position marked by crosses in (c). Inset is the d*I*/d*V* spectrum collected on the Au(111) surface, and the position is marked by a blue cross in (a). e) BPGN with 1 ML K deposited on the hollow sites (V_S_ = 3.5 V, I = 50 pA). f) Different patterns from the same area of type‐I K layer under the sample bias of 3.5 V (up) and ‐1 V (down). The periodicity of the type‐I K is identified as 1.45 nm.

The BPGN layers were prepared on Au(111) surface by molecular beam epitaxial deposition, as shown in **Figure** **1**a. Scanning tunneling microscopy (STM) images of BPGN in Figure 1b manifests that each corner of the honeycomb cell is consisted of a trimer‐like protuberance, corresponding to the up‐buckled phosphorus atoms in the P_9_ subunit.^[25^ ^]^ The lattice periodicity of BPGN is measured to be 1.45 nm, matching well with its atomic lattice structure. The adjacent P_9_ subunits are connected with each other by three gold atoms, forming a hollow site in the center and two bridging sites between the three collinearly connected gold atoms, as indicated in Figure 1b. After deposition of potassium atoms on BPGN, they were prior to occupy the hollow sites at low coverage (<1 monolayer, ML), which are named as type‐I K. Such absorption sites were further confirmed by the STS mapping and topographical images of type‐I K on BPGN under different sample bias, as shown in Figures S1 and S2 (Supporting Information). A region containing 0.7 ML type‐I K was chosen to investigate the electronic state on the surface, as depicted in Figure 1c. Two d*I*/d*V* spectra were collected on the local type‐I K layers and underlying BPGN surface, as marked by red and black crosses, respectively. Features of small dip and hump are observed in both spectra near −0.5 V as marked by arrows in Figure 1d. We found this electronic state possesses similar energy and features to Shockley surface state^[^ ^26^ ^]^ (SSS) taken on Au(111) substrate (the inset in Figure 1d). It was reported that the SSS can survive on silicon intercalated BPGN on Au(111),^[^ ^27^ ^]^ because SSS does not hybridize to BPGN. Thus, the dip and hump feature in our experiment is also attributed to SSS from Au(111) substrate. In addition, it suggests that this 2DEG does not vanish after formation of BPGN and type‐I K layers. Figure 1e shows atomic structure of BPGN covered by 1 ML K atoms. Notably, in contrast to the positive sample bias, the K atoms are invisible at negative sample bias during the STM measurements, as shown in Figure 1f. Such a phenomenon indicates that K atoms might donate their electrons to the substrate, and as a result, only manifest themselves in STM image under the positive sample bias.^[28^ ^]^

**Figure 2:**
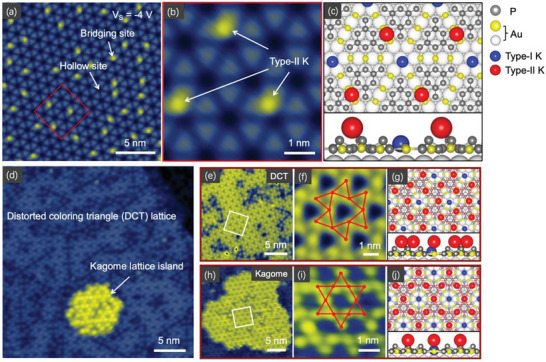
DCT and kagome lattices imaged by STM. a) Small amount of 2nd layer K deposited randomly on the substrate (V_S_ = −4.0 V, I = 400 pA). b) Close‐up image in the red square in (a). c) Schematic of the atomic structure of type‐II K atoms absorbed in the middle of two connected Au atoms. Up and down panel correspond the top and side view, respectively. d) Large area of distorted colouring triangle lattice (DCT) and a small kagome lattice island (V_S_ = 0.3 V, I = 100 pA). e–g) Large region image, close‐up atomically resolved STM image and schematic of atomic structure of DCT lattice (V_S_ = 0.2 V, I = 50 pA). h–j) Large region image, close‐up atomically resolved STM image and schematic of atomic structure of kagome lattice (V_S_ = 0.3 V, I = 100 pA). The real red lines in (f) and (i) and dot red lines in (g) and (j) are a guide to the eye to identify the periodic lattice.

At higher coverage than 1 ML, K atoms began to reside randomly at the bridging sites, as shown in **Figure** **2**a. We name K atoms at bridging site as type‐II K. Figure 2b,c exhibit the high‐resolution STM image and the schematic of atomic structure. As the coverage increases to 2 ML, two distinct K lattices, DCT and kagome, formed simultaneously on the surface. We found the main area is consisted of DCT lattice, while a few small islands in kagome can been seen, as shown in Figure 2d. The atomically resolved image of DCT and kagome lattices are shown in Figure 2f,i, respectively. Their atomic structures are shown in Figure 2g,j, in which both DCT and kagome lattices are represented by the red atoms connected by dot lines. The distance between NN K atoms in DCT lattice is *a*  =  7.71 Å, which is very close to the value of 7.16 Å of kagome lattice. The structural evolution from DCT to kagome lattice can be achieved by the unitary transfer of resident sites on the substrate, that is, moving every K atom from bridging sites to the top of the medially connected Au atoms (top sites). In order to understanding the nature of structural evolution, DFT calculation is carried out to evaluate the energy of formation (EOF) for the two lattices. The results manifested that the kagome lattice has a higher EOF than DCT lattice (Table S1, Supporting Information). This result was confirmed experimentally, in which increased number of kagome islands formed upon substrate temperature increased during deposition. As a result, the kagome lattice is a meta‐stable phase compared with the DCT lattice on the BPGN substrate, which in return explains why DCT lattice has a higher yield of synthesis in our MBE experiments. On another hand, from the perspective of symmetry, the six‐fold rotational symmetry undergoes a spontaneous breaking to three‐fold rotational symmetry, corresponding to the evolution from kagome to DCT lattice. This breaking of rotational symmetry is also reflected by the apparent height distortion of kagome lattice, as depicted in Figure S5 (Supporting Information), which is caused by the spontaneous trend of sliding from top sites to bridging sites of K atoms. In a more general sense, the lower the symmetry of one system, the more stable the structure.^[29^ ^]^ Owing to the low symmetry of DCT lattice, we expect that more robust electronic properties can be observed in this system.

**Figure 3:**
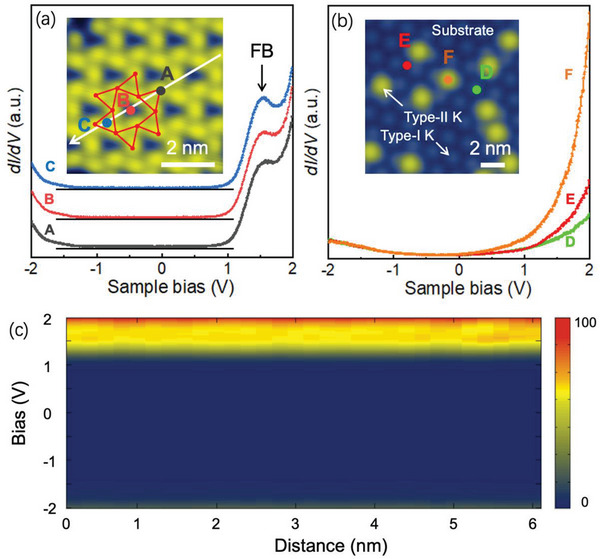
Electronic structure of DCT lattice. a) STS spectra acquired on the DCT lattice show FB peaks: an emergent peak at ≈1.5 V. The inset shows the position of A, B and C in STS spectra (V_S_ = −0.1 V, I = 200 pA). Spectra are offset vertically for clarity and the underline below each spectrum represents the zero DOS level. b) STS spectra acquired at D, E and F. The inset shows the selected point sites (V_S_ = 3.5 V, I = 50 pA). c) STS spectra acquired along the white arrow in the inset of (a).

To explore the nature of electronic structure, we conducted d*I*/d*V* spectra on the surface of DCT lattice. A gap‐like feature with a nearly zero DOS ranging from −1.2 to 0.6 V were found in **Figure** **3**a, in which the underlines represent the zero‐value level for each d*I*/d*V* spectrum. Especially, the spectra collected at three representative positions marked as A (corner site), B (center site) and C (corner center site) in DCT lattice in the inset were found to share a common feature with a peak at ≈1.5 V. To explore the possible origin of this peak, we collected local d*I*/d*V* tunneling spectra from the substrate with random type‐II K atoms absorbed on it, as shown in Figure 3b. Point D, E and F in the inset represent the site of the middle between two type‐I K atoms, top of one type‐II K atom and top of one type‐I K atom, respectively. No obvious features were found in the spectra and the peaks around the sample bias of 1.5 V disappeared. The non‐local property of this state was further revealed by the STS spectra acquired along the line in the inset of Figure 3a, which exhibit a robust peak position near 1.5 V, as revealed in Figure 3c. Such results indicate that the observed peaks do not originate from the underlying substrate or localized states caused by individual type‐II K atoms on it. It suggests that this electronic state high possibly derives from the modulation of surface state by the stable DCT lattice.

**Figure 4:**
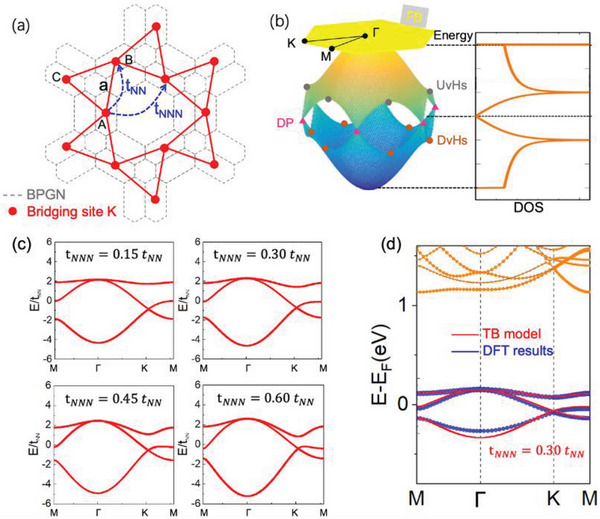
TB and DFT calculations of electronic DCT lattice. a) Schematic of the DCT lattice on BPGN and the hopping parameter in TB model. b) A k‐space dispersion of the electronic bands and the corresponding DOS of the DCT lattice, calculated using the TB model for the only NN hopping considered. The energy bands are identical with kagome bands, with FB, up van Hove singularities (UvHs), down van Hove singularities (DvHs) and Dirac points (DP) marked in the first Brillouin zone. c) Energy bands calculated by TB model with different values of *t_NNN_*/*t_NN_*. d) Energy bands calculated by DFT method and the parameter of *t_NNN_* =  0.30 *t_NN_* in TB model is chosen to fit the results.

In order to shed light on the physical origin of the observed STS peaks in Figure 3a, we performed density functional theory (DFT) calculations combined with TB model analysis. The 2DEG feature of the surface was supported by our DFT calculations, in which the DCT lattice was placed on the BPGN and Au(111) substrate, as shown in Figure S6 (Supporting Information). As a result, our TB model is simplified as a DCT lattice modulating the 2DEG. The TB model involves three K atoms in one unit cell marked as A, B and C, as shown in **Figure** **4**a. The nearest neighboring (NN) and the next‐nearest neighboring (NNN) electron hopping strength between atomic orbitals associated to lattice sites are denoted as *t_NN_* and *t_NNN_*, respectively. The TB Hamiltonian in second quantization form reads:

(1) $$H\;=\sum_{\langle i,\;j\rangle }t_{NN}c_{i}^{†}c_{j}\;+\sum_{\ll i,\;j\gg }t_{NNN}c_{i}^{†}c_{j}+H.c.$$

where $c_{i}^{†}$ and *c_j_* are the electron creation and annihilation operator at site *i* and *j*, respectively. Here 〈*i*,  *j*〉 denotes NN hopping path and ≪ *i*,  *j* ≫ denotes NNN hopping path. When only NN hopping is considered, the electronic structure manifests three bands with the appearance of a flat band, together with two Dirac points and van Hove singularities (vHs) in the reciprocal space, as shown Figure 4b. In principle, the feature of the d*I*/d*V* spectrum or DOS corresponding to a FB should be a narrow and sharp peak.^[^ ^17^, ^30^ ^]^ However, the peak ≈1.5 V observed in Figure 3a is fairly broad, which is attributed to the non‐negligible NNN hopping progress. The NNN hopping strength should be strongly affected by the distance between NNN K atoms and the anisotropic potential field caused by the asymmetric atomic environment. In the above DCT lattice, the distance between NNN K atoms is 9.68 Å, which is only 20% longer than the distance between NN K atoms. On the other hand, the asymmetric shape of electronic distribution should conversely enhance the coupling between NN K atoms, for their shape is along the NN hopping direction. A series of band structures calculated by TB model corresponding to different ratios between *t_NNN_* and *t_NN_* are presented in Figure 4c. It is clearly found that the FBs gradually become dispersive as *t_NNN_* increases. The TB calculation details can be found in the Supplemental Material (Figure S7, Supporting Information). Figure 4d presents the DFT calculations with all hopping strength considered in DCT lattice and a nearly flat band turns out to be found. By fitting the DFT results, it is found that $\frac{t_{NNN}}{t_{NN}}=\;0.3$ is a proper value in our TB model. To enhance the reliability of our model, we calculated the wavelength of the plane wave on Au(111) surface corresponding to the energy level of 1.5 eV above Fermi surface, and compared it to the periodicity of DCT potential field. According to the previous reports, the wave vector (*k*) of such a single free electron is ≈ 0.4 Å^−1^,^[^ ^31^ ^]^ whose wavelength (λ) can be calculated by $\lambda \;=\frac{2\pi }{k}\;=15.7$ Å. This value is very close to the double distance between adjacent K atoms in DCT lattice, that is 2a = 15.42 Å. As a result, the physical progress corresponding to the peaks ≈1.5 V in Figure 3a can be described as the destructive interference of plane waves between the DCT lattice sites, which eventually results in the localization of electronic state within the center hexagon and causes a FB.

## 3 Conclusion

In summary, we have experimentally realized a stable‐structure electronic DCT lattice with the emergence of an electronic FB on a 2D K lattice on BPGN surface. The growing process of the DCT lattice has been presented in detail and the electronic properties of the substrate and DCT lattice have been characterized by STS technique. The combination of DFT calculations and TB model reveals that the emergent FB peaks in STS are given rise to the destructive phase interference of 2DEG on Au(111) surface in the potential field of DCT lattice. Besides, the potential valleys on the BPGN substrate provide an ideal platform for absorbing other different atoms. For example, magnetic elements are expected to be trapped in the valleys to explore strong correlated phenomena, such as frustrated magnetism^[^ ^32^ ^]^ and spin ice state.^[33^ ^]^ The realization of electronic DCT lattice in our experiments will open a new route to explore a variety of exotic many‐body phenomena originating from FB physics.

## 4 Experimental Section

### Sample Preparation

Au(111) substrate was first prepared by 1 keV Ar^+^ ion sputtering at room temperature and followed by annealing at 850 K for several cycles. Then, P atoms were deposited on the surface under ultra‐high vacuum (UHV) conditions from a heated well‐degassed Ta wafer. The Au(111) substrate temperature was maintained at 473 K during the deposition process and the deposition flux was ≈0.05 monolayer per minute. Then, K atoms were deposited on the BPGN under UHV conditions from a heated well‐degassed K source. The overall deposition time of 2 ML K atoms in experiments had exceeded 1 h.

### STM Measurements

All the STM images were acquired in constant current mode at liquid helium temperature (4.2 K) by using a low‐temperature (LT) UHV scanning tunneling microscopy from Unisoku Co., Ltd. (USM 1500). The STS differential conductance spectra (d*I*/d*V*) were obtained with standard lock‐in detection by applying a small amplitude modulation on the sample bias voltage (10 mV, 973 Hz). All STM images were processed using WSxM software.^[34^ ^]^

### DFT Calculations

DFT calculations were performed within the framework of density functional theory with ab initio pseudopotentials and plane‐wave formalism as implemented in the Vienna ab initio simulation package (VASP).^[35^ ^]^ The Brillouin zone was integrated with a Γ‐centered 3×3×1 k‐mesh. The plane‐wave cut‐off energy was set as 400 eV. The structures were relaxed until the remaining force acting on each atom is less than 0.01 eV Å^−1^ within the generalized gradient approximation (GGA) with the Perdew‐Burke‐Ernzerhof (PBE) functional.^[36^ ^]^ The Au(111) surface was simulated by a four‐layer slab model, and the bottom two layers of Au atoms were fixed. A vacuum layer of ≈15 Å was applied to eliminate the interaction between neighbor slabs. The STM simulation conducted based on the Tersoff‐Hamann method, and the energy range were −0.9–−1.1 eV and 3.4 – 3.6 eV respectively, corresponding to the experimental values.

## Conflict of Interest

The authors declare no conflict of interest.

## Supporting information

Supporting Information

## Data Availability

The data that support the findings of this study are available from the corresponding author upon reasonable request.
