# Supplementary material for: Electronic Flat Band in Distorted Colouring Triangle Lattice
Source: Adv Sci (Weinh). 2023 Oct 15;11(37):2303483. doi: 10.1002/advs.202303483 (PMC11462289; doi:10.1002/advs.202303483)
Supplement: Supplementary file 1 — Supporting Information [file ADVS-11-2303483-s001.pdf]

ADVANCED SCIENCE
Open Access

Supporting Information

for *Adv. Sci.*, DOI 10.1002/advs.202303483

Electronic Flat Band in Distorted Colouring Triangle Lattice

Yaqi Li, Shuwei Zhai, Yani Liu, Jingwei Zhang, Ziyuan Meng, Jincheng Zhuang, Haifeng Feng, Xun Xu, Weichang Hao*, Miao Zhou*, Guang-Hong Lu, Shi Xue Dou and Yi Du**

Supporting information for

Electronic flat band in distorted colouring triangle lattice

Yaqi Li[1,5], Shuwei Zhai[1], Yani Liu[2], Jingwei Zhang[1,5], Ziyuan Meng[1,5], Jincheng Zhuang[1,5], Haifeng Feng[1,5], Xun Xu[3], Weichang Hao[1,5*], Miao Zhou[1,6*], Guang-Hong Lu[1,7], Shi Xue Dou[4] and Yi Du[1,5*]*

1 School of Physics, Beihang University, Haidian District, Beijing 100191, China.

2 Institute of Physics, Chinese Academy of Sciences, Beijing 100190, China.

3 Institute for Superconducting and Electronic Materials, Australian Institute for Innovative Materials, University of Wollongong, Wollongong, New South Wales 2500, Australia.

4 Institute of Energy Materials Science, University of Shanghai for Science and Technology, Yangpu District, Shanghai 200093, China.

5 Centre of Quantum and Matter Sciences, International Research Institute for Multidisciplinary Science, Beihang University, Beijing 100191, China.

6 Beihang Hangzhou Innovation Institute Yuhang, Hangzhou 310023, China.

7 Beijing Key Laboratory of Advanced Nuclear Materials and Physics, Beihang University, Beijing, 100191, China.

Yaqi Li, Shuwei Zhai and Yani Liu contributed equally to this work.

* Correspondence authors. E-mail: yi_du@buaa.edu.cn; mzhou@buaa.edu.cn; whao@buaa.edu.cn; jincheng@buaa.edu.cn

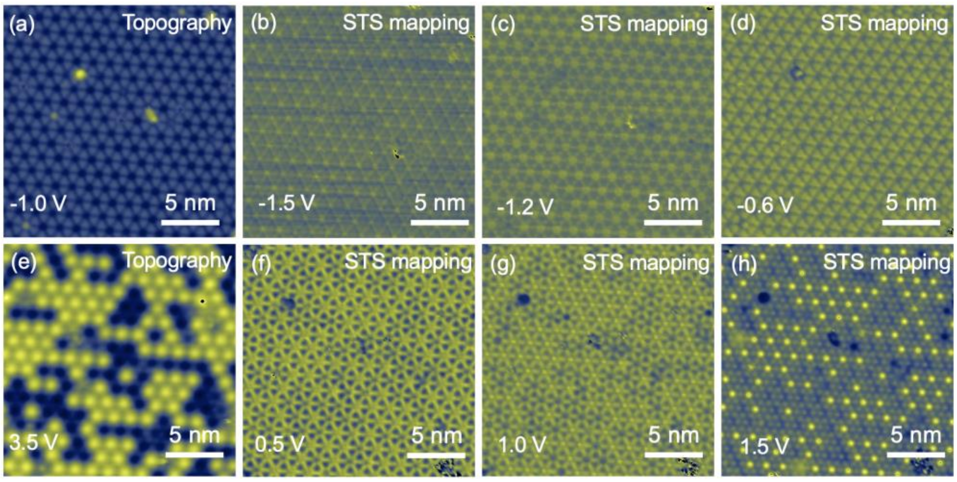

Fig. S1 0.7 ML Type-I K covered BPGN and the corresponding STS mapping under different sample bias. (a) and (e) Topography of 0.7ML K covered BPGN under the sample bias of -1.0 V and 3.5 V, respectively. The K atoms are invisible under a negative sample bias in (a). (b-d) STS mapping images of the occupied states under the sample bias of -1.5 V, -1.2 V and -0.6 V, respectively. (f-h) STS mapping images of the unoccupied states under the sample bias of 0.5 V, 1.0 V and 1.5 V, respectively. The states of K atoms were gradually appearing at hollow sites as sample bias increased from negative to positive values.

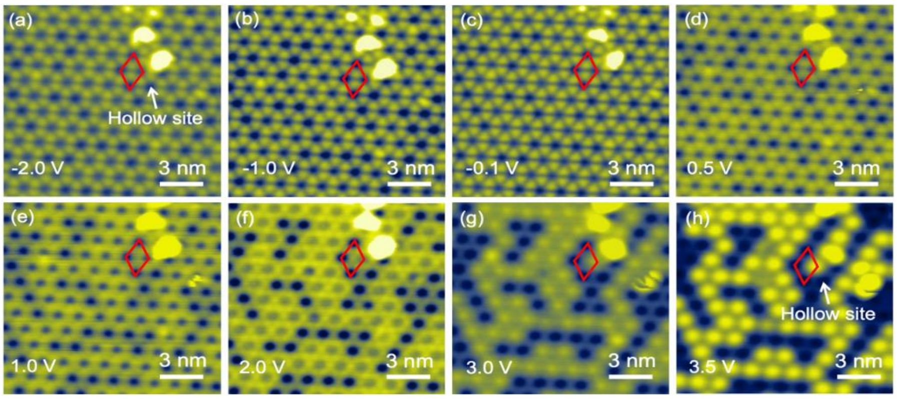

Fig. S2 Topographical images of type-I K on BPGN under different sample bias. (a-h) exhibit the topographical images of Type-I K on BPGN in the same area under

both negative and positive sample bias in which the red rhombuses mark the unit cell of BPGN. The type-I K is invisible when the sample bias is lower than 2.0 V (a-f). While the sample bias reaches 3.0 V and 3.5 V, the type-I K atoms can be clearly distinguished at the hollow sites on BPGN, as shown in (g) and (h), respectively.

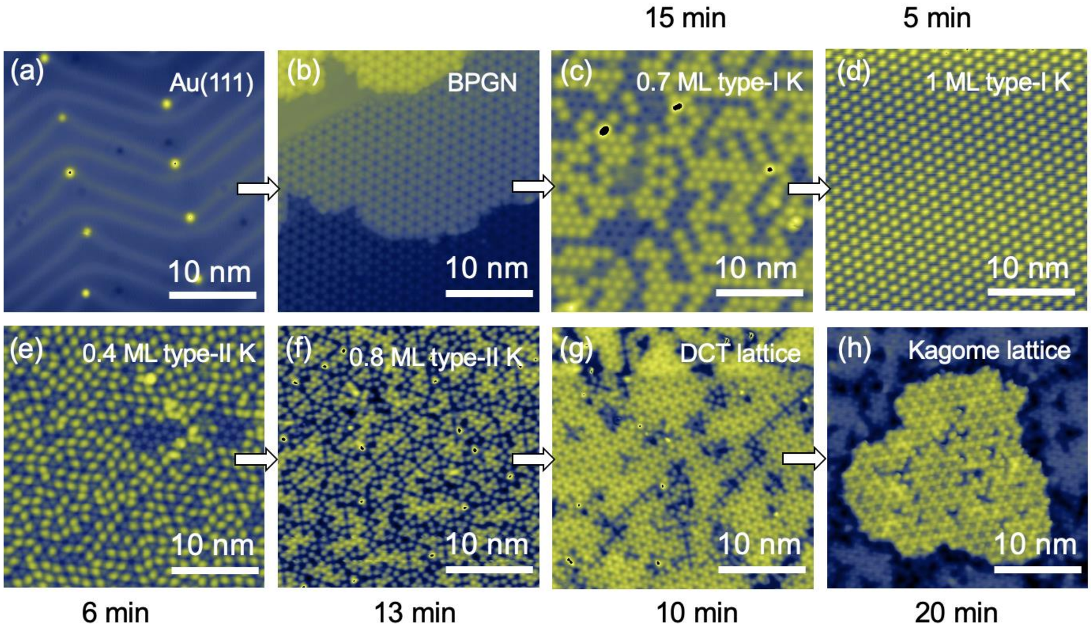

Fig. S3 An overall growing progress of K atoms on BPGN and Au(111) substrate. (a) Clean Au(111) surface with 22×3 reconstruction on it. (b) 1 ML BPGN grown on Au(111) substrate. (c-d) 0.7 ML and 1 ML type-I K atoms grown for 15 minutes and 5 minutes at the hollow site, respectively. (e) 0.4 ML type-II K grown for 6 minutes. (f) 0.8 ML type-II K grown for 13 minutes with a short-range ordered pattern with the periodicity of $\sqrt{3} \times \sqrt{7}$ with respect to BPGN layers. (g) Large area of DCT lattice grown for 10 minutes. (h) Kagome island grown for 20 minutes.

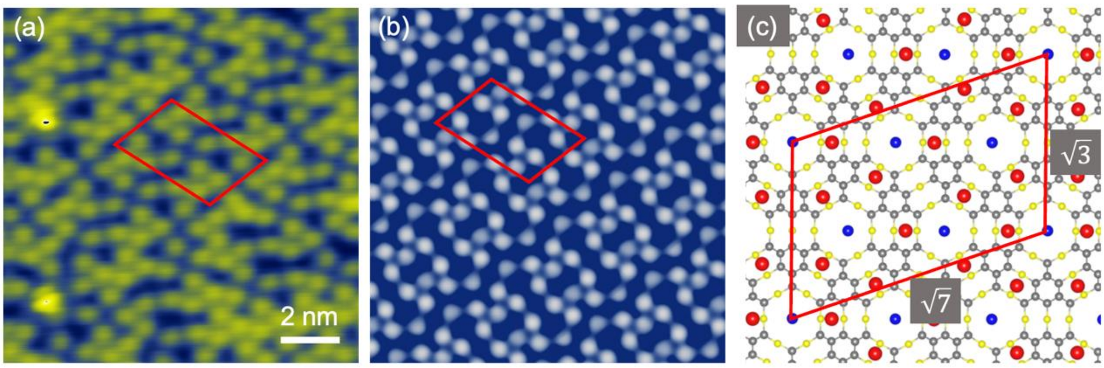

Fig. S4 $\sqrt{3}\times\sqrt{7}$ structure of type-II K. (a-c) STM image, simulated STM image and the atomic structure of $\sqrt{3}\times\sqrt{7}$ pattern, respectively.

Stage	Energy of formation ( )
1 ML type-I K	-2.94 eV
0.33 ML type-II K	-2.53 eV
0.8 ML type-II K (√3×√7 structure)	-2.42 eV
1 ML type-II K (DCT lattice)	-2.08 eV
1 ML type-II K (Kagome lattice)	-1.90 eV

Table. S1 Energy of formation ($E_{formation}$) of structures at different growing stages. Five growing stages were chosen to compare their $E_{formation}$, which is defined as

$$E_{formation} = \frac{E(sub + layer) - E(sub) - E(layer)}{Number\ of\ K\ atom}$$

Note that $E_{formation}$ is growing when the coverage of K is increasing from the second stage to the fourth stage. However, Kagome lattice shares the same coverage with the DCT lattice but has a higher $E_{formation}$, which indicates that DCT lattice is more stable than Kagome lattice.

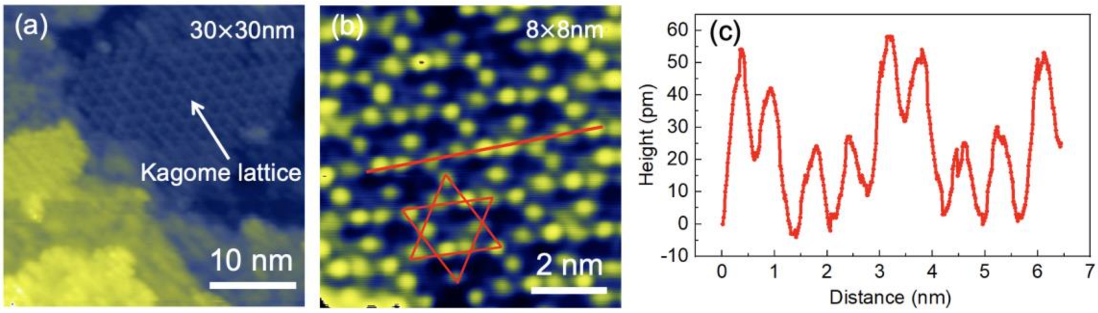

Fig. S5 Kagome lattice with large height distortion. (a) large area of Kagome lattice was acquired successfully when the temperature of the substrate is increased. (b) Close-up STM image of Kagome lattice with a height distortion. (c) Measure of height difference of K atoms in Kagome lattice along the line in (b), in which the height distortion is about 30 pm.

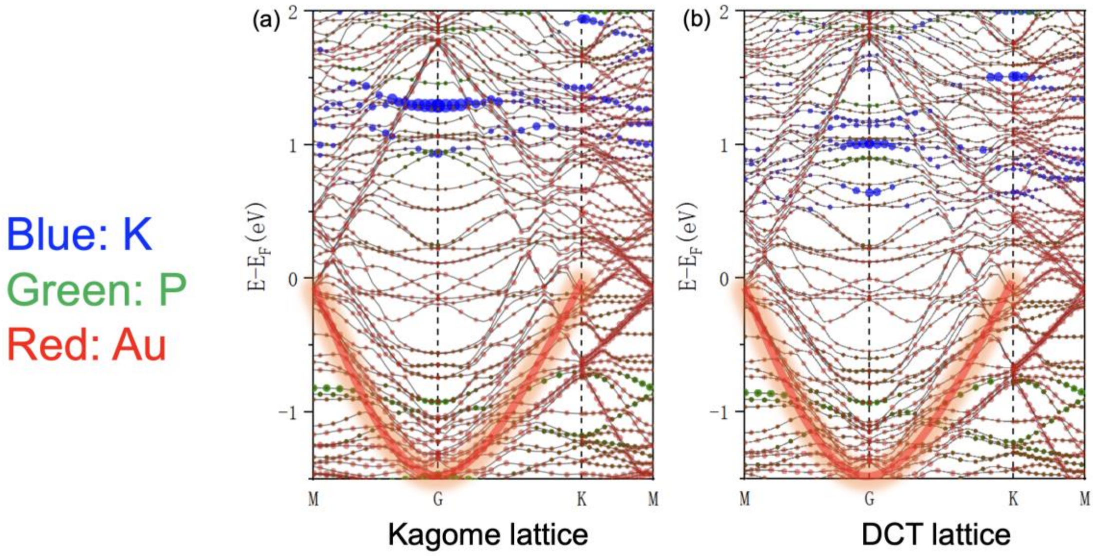

Fig. S6 DFT calculation of kagome and DCT lattices. (a-b) DFT calculation of Kagome lattice and DCT lattice on BPGN/Au(111) substrate, respectively. The FBs in two lattices are heavily hybridized with Au and P orbits. However, the surfaces exhibit a large background of 2DEG from Au(111) surface state, as indicated by the red light para-curves.

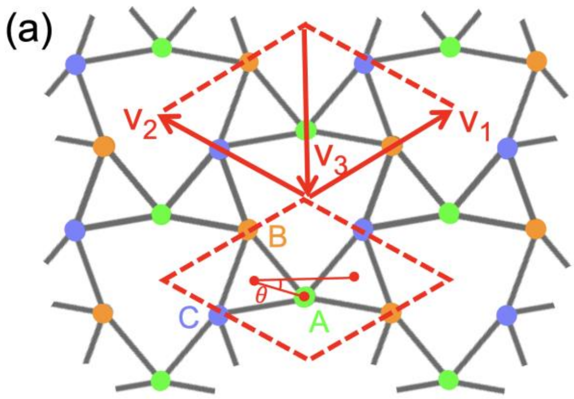

Fig. S7 TB model calculation of DCT lattice. (a) schematic of TB model calculation. Red dot lines depict the unit cell of DCT lattice. Theta is calculated as 21.1°.

When NN hopping and NNN hopping are both considered, the Hamiltonian can be written as:

$$H^{DCT}(\vec{k}) = \begin{bmatrix} 0 & H_{12} & H_{13} \\ H_{12}^{*} & 0 & H_{23} \\ H_{13}^{*} & H_{23}^{*} & 0 \end{bmatrix},$$

where

$$H_{12} = -2t_{NN}cos\left(\vec{k}\cdot\frac{\vec{v_3}}{2}\right)e^{\frac{\sqrt{3}tan\theta}{6}i\vec{k}\cdot(\vec{v_1}-\vec{v_2})} - t_{NNN}e^{\frac{\sqrt{3}-tan\theta}{2\sqrt{3}}i\vec{k}\cdot(\vec{v_2}-\vec{v_1})}$$

$$H_{13} = -2t_{NN}cos\left(\vec{k}\cdot\frac{\vec{v_2}}{2}\right)e^{\frac{\sqrt{3}tan\theta}{6}i\vec{k}\cdot(\vec{v_1}-\vec{v_3})} - t_{NNN}e^{\frac{\sqrt{3}-tan\theta}{2\sqrt{3}}i\vec{k}\cdot(\vec{v_3}-\vec{v_1})}$$

$$H_{23} = -2t_{NN}cos\left(\vec{k}\cdot\frac{\vec{v_1}}{2}\right)e^{\frac{\sqrt{3}tan\theta}{6}i\vec{k}\cdot(\vec{v_2}-\vec{v_3})} - t_{NNN}e^{\frac{\sqrt{3}-tan\theta}{2\sqrt{3}}i\vec{k}\cdot(\vec{v_3}-\vec{v_2})}$$

and H^{*} means the Hermite conjugation of H.

When only NN hopping is considered, diagonalization of the Hamiltonian gives rise to the energy bands, which can be expressed as:

$$E_0 = 2t_{NN};\ E_{\pm}(\vec{k}) = -t_{NN} \pm t_{NN}\sqrt{8\cos\left(\vec{k}\cdot\frac{\vec{v_1}}{2}\right)\cos\left(\vec{k}\cdot\frac{\vec{v_2}}{2}\right)\cos\left(\vec{k}\cdot\frac{\vec{v_3}}{2}\right)+1},$$

which is identical with Kagome bands.
